# Does genetic structure reflect differences in non-breeding movements? A case study in small, highly mobile seabirds

**DOI:** 10.1186/s12862-017-1008-x

**Published:** 2017-07-05

**Authors:** Petra Quillfeldt, Yoshan Moodley, Henri Weimerskirch, Yves Cherel, Karine Delord, Richard A. Phillips, Joan Navarro, Luciano Calderón, Juan F. Masello

**Affiliations:** 1 Department of Animal Ecology and Systematics, Justus Liebig University Giessen, Heinrich-Buff-Ring 38, 35392 Giessen, Germany; 2 Department of Zoology, University of Venda, Private Bag X5050, Thohoyandou, 0950 Republic of South Africa; 3 Centre d’Etudes Biologiques de Chizé, UMR 7372 CNRS-Université de La Rochelle, 79360 Villiers-en-Bois, France; 4 British Antarctic Survey, Natural Environment Research Council, High Cross, Madingley Road, Cambridge, CB3 0ET UK; 5 Department of Conservation Biology, Estación Biológica de Doñana (EBD-CSIC), Avda. Américo Vespucio s/n, 41092 Seville, Spain

**Keywords:** Falkland / Malvinas Islands, Genetic structure, Kerguelen Islands, Non-breeding distribution, Phylogeography, Procellariidae, South Georgia, Spatial distribution

## Abstract

**Background:**

In seabirds, the extent of population genetic and phylogeographic structure varies extensively among species. Genetic structure is lacking in some species, but present in others despite the absence of obvious physical barriers (landmarks), suggesting that other mechanisms restrict gene flow. It has been proposed that the extent of genetic structure in seabirds is best explained by relative overlap in non-breeding distributions of birds from different populations. We used results from the analysis of microsatellite DNA variation and geolocation (tracking) data to test this hypothesis. We studied three small (130–200 g), very abundant, zooplanktivorous petrels (Procellariiformes, Aves), each sampled at two breeding populations that were widely separated (Atlantic and Indian Ocean sectors of the Southern Ocean) but differed in the degree of overlap in non-breeding distributions; the wintering areas of the two Antarctic prion (*Pachyptila desolata*) populations are separated by over 5000 km, whereas those of the blue petrels (*Halobaena caerulea*) and thin-billed prions (*P. belcheri*) show considerable overlap. Therefore, we expected the breeding populations of blue petrels and thin-billed prions to show high connectivity despite their geographical distance, and those of Antarctic prions to be genetically differentiated.

**Results:**

Microsatellite (at 18 loci) and cytochrome *b* sequence data suggested a lack of genetic structure in all three species. We thus found no relationship between genetic and spatial structure (relative overlap in non-breeding distributions) in these pelagic seabirds.

**Conclusions:**

In line with other Southern Ocean taxa, geographic distance did not lead to genetic differences between widely spaced populations of Southern Ocean petrel species.

**Electronic supplementary material:**

The online version of this article (doi:10.1186/s12862-017-1008-x) contains supplementary material, which is available to authorized users.

## Background

The Southern Ocean (water masses south of the Subtropical Front) is characterized by an environmental gradient from subantarctic to polar waters, and the fauna reflect adaptations to the thermal and latitudinal structure in this region (e.g. [1, 2]). Seabirds in the Southern Ocean are faced with a limited availability of habitable islands, resulting in disjunctive breeding distributions, with populations often located at island groups that are several thousand kilometres apart [3]. Because geographical barriers are absent across the Southern Ocean, physical isolation by geographic landmarks may not restrict gene flow. However, distance as well as intrinsic barriers, such as site fidelity, can reduce dispersal and lead to genetic structuring of seabird populations [4, 5]. Geographical distance can result in local differentiation (isolation by distance), the extent of which increases as connectivity decreases [4, 6]. However, the movements of pelagic seabirds, which are extensive during the non-breeding season, can counteract the geographical isolation of their breeding colonies. Hence, integrative studies of both population genetic structure and at-sea distributions are required to understand evolutionary processes in these taxa. An overlap of nonbreeding areas may facilitate contact and pair-bonds between individuals of different populations, even when their breeding areas are far apart. A direct study of site fidelity and movements between colonies in these species, e.g. using mark-recapture techniques, is difficult due to the remoteness and size of the breeding colonies, which can be as large as two million pairs (e.g. Thin-billed prions *Pachyptila belcheri* on New Island, Falkland Islands). Thus, genetic methods are required.

Several studies that have analysed patterns of genetic diversity in seabird species with circumpolar distributions in the Southern Ocean, have found little genetic and phylogeographic structure despite the large distances between colonies (e.g. Adélie penguins *Pygoscelis adeliae*, [7]; grey-headed albatrosses *Thalassarche chrysostoma*, [8]; wandering albatrosses *Diomedea exulans*, [9]). In contrast, the black-browed albatross *T. melanophris* displays genetic structure despite the absence of physical barriers, potentially because of its stronger association with continental shelf habitats; this conclusion was based on the comparison with the closely-related grey-headed albatross, which forages mainly at frontal systems which are circumpolar [8]. Similarly, genetic structure has been found in other species associated with foraging on land or over continental shelves (giant petrels [10]; skuas [11]). Alternatively, in white-chinned petrels the Atlantic and Indian Ocean populations are not separated genetically, but New Zealand forms a separate cluster [12]. To explain this variability, Friesen et al. [4] suggested that the extent of genetic structure in seabirds could be explained by the overlap of non-breeding areas, as most species with two or more population-specific non-breeding areas showed phylogeographic structure.

Due to their high mutation rates, microsatellite loci (i.e. regions across the genome where short nucleotide sequences are repeated in tandem) provide powerful tools in population genetics. They allow the testing of hypotheses related to within- and between-population genetic variability and the estimation of other demographic parameters such as gene flow and effective population size [13, 14]. Recently, Moodley et al. [15] used next-generation sequencing to develop microsatellite markers for thin-billed prions (*Pachyptila belcheri*), and other *Pachyptila* species (Aves, Procellariiformes; albatrosses and petrels). Here we use this newly developed marker set, together with tracking (geolocation) data to test the hypothesis that genetic structure is dependent on the non-breeding distribution pattern in thin-billed prions, Antarctic prions *Pachyptila desolata* and the closely-related blue petrel *Halobaena caerulea*. These small petrels have wide breeding distributions, including islands in different ocean basins, and are wide-ranging from subtropical to Antarctic waters [2, 16–19], making them ideal biological models to test for a correspondence between genetic structure and non-breeding distribution. The aim of the present study was thus to test the hypothesis that the extent of genetic structure is explained by the degree of overlap in non-breeding distributions of birds from different populations, by comparing three closely-related species with different migration strategies. We expected higher gene flow in the species in which populations showed greatest overlap during the non-breeding season.

## Methods

### Study species and sample collection

Blue petrels, thin-billed prions and Antarctic prions breed on islands in the Southern Ocean, and in each species, we sampled two large breeding populations, one in the south-west Atlantic Ocean (Falkland Islands and South Georgia) and the other in the Indian Ocean (Kerguelen Islands) [20]. The three petrel species migrate away from their breeding grounds during the non-breeding season, where they segregate by choosing temperate (Antarctic prions), subantarctic (thin-billed prions) or polar (blue petrels) waters [2, 17–19]. Blue petrels, thin-billed prions and Antarctic prions show the typical procellariiform pattern of high longevity (several decades), and an extended period (several years) before they return to the colony to breed [21]. Both sexes take part in incubation, and in feeding of their single, slow-growing chick [22, 23]. These three species are zooplanktivorous, with a preference for crustaceans [24, 25].

During 2010–2012, blood samples were obtained from adult birds breeding in colonies of thin-billed prions located at Île Mayès, Kerguelen (49°28′S, 69°57′E; *N* = 34) and New Island, Falkland/Malvinas Islands (51°43′S, 61°18′W; *N* = 42); Antarctic prions at Île Verte, Kerguelen (49°30′S, 70°02′E; *N* = 38) and Bird Island, South Georgia (54°00′S, 38°02′W; *N* = 35); and blue petrels at Île Mayès (*N* = 30) and Bird Island (*N* = 19). Blood (0.2–0.4 ml) was sampled by puncture of the wing vein and collection using heparinized capillaries, or using 0.3 ml syringes, and immediately transferred to FTA classic cards (Whatman International Ltd). These breeding colonies represent major breeding sites of all three species, with estimates at each site of several million birds for prions, and >100,000 individuals for the blue petrel [20, 26].

### Analyses of distribution

Data on non-breeding distributions were obtained using 1 g leg-mounted geolocators, or Global Location Sensors (GLS loggers; model MK10, supplied by British Antarctic Survey and Biotrack UK), as described previously [2, 17, 18, 27]. Briefly, we attached geolocators to 12 to 25 individuals from each population during the breeding seasons 2009/10 (New Island, Bird Island) and 2011/12 (Kerguelen). Repeatability of migration routes across years is high in these species [27]. The geolocators weighed 1 g, equivalent to <1% of the mean body mass of blue petrels (ca. 190 g), thin-billed prions (ca. 130 g) and Antarctic prions (ca. 160 g), and were fixed to plastic leg bands. Tagged individuals were fitted with standard metal rings on the other leg. Burrows were revisited and devices retrieved in the following two seasons (New I., *N* = 20 thin-billed prions; Bird I., *N* = 9 Antarctic prions, *N* = 11 blue petrels; Kerguelen, *N* = 10 Antarctic prions, *N* = 16 blue petrels and *N* = 19 thin-billed prions). We analysed distribution data from one non-breeding season per bird. Processing of geolocator data was carried out as described previously [18]. Changes in distribution by month were examined using kernel analysis of filtered locations [28]. The non-parametric fixed kernel density estimator was used to determine the 95% density contour. Kernel densities do not require serial independence of observations when estimating foraging ranges [29]. Kernel analyses were performed in a Lambert azimuthal equal-area projection centred on the South Pole using ARCGIS 9.3 (ESRI, Redlands, CA, USA) using HAWTH’S ANALYSIS TOOLS [30]. The overlap in 95% density polygons between populations were calculated for each species using the INTERSECT and UNION tools of ARCGIS 9.3.

### Genetic analyses

Genomic DNA was extracted from FTA cards as described in Merino et al. [31]. We genotyped all 198 adults at 25 microsatellite loci developed for thin-billed prions, and shown to be highly informative for Antarctic prions and blue petrels [15]. As in our previous study [15], genotypes were assigned with GENEMARKER 1.85 (SoftGenetics LLC, State College, PA, USA). Twenty percent of the samples were re-scored by a separate researcher, with an error rate of <5%. We used these cases to define our threshold for scoring as “missing data”. We screened the microsatellite data for the presence of null alleles. In a previous study [15], we showed using MICROCHECKER 2.2.3 [32] that the average frequency of null alleles in our data set was low. Measures of genetic diversity were estimated using MSA 4.05 [33] and ARLEQUIN 3.5 [34]: number of alleles per locus (A), observed heterozygosity (Ho) and expected heterozygosity (He). The probability of deviation from Hardy–Weinberg equilibrium (HWE) and the inbreeding coefficient (*F*_*IS*_) and its significance were calculated for each population using GENEPOP (Table 1) [35].

**Table 1:** Genetic variation and tests for selection and/or population expansion. Parameters are based on 18 polymorphic microsatellites recorded in Atlantic and Indian Ocean populations of the blue petrel *Halobaena caerulea*, thin-billed prion *Pachyptila belcheri*, and Antarctic prion *P. desolata*

Species	Site	Microsatellites						Mitochondrial DNA cytochrome *b* 889 bp	
		*N*	*A*	*H*_*o*_	*H*_*e*_	*F*_*IS*_	*N*	*D*	*F*_*S*_
Blue petrel	South Georgia	19	7.2 ± 3.7	0.53 ± 0.24	0.66 ± 0.28	0.206^***^	12	−1.385^ns^	−1.088^ns^
	Kerguelen Islands	30	7.7 ± 4.9	0.52 ± 0.28	0.62 ± 0.30	0.168^***^	15	−1.159^ns^	−0.649^ns^
Thin-billed prion	Falkland Islands	42	8.4 ± 2.6	0.71 ± 0.10	0.74 ± 0.11	0.042^ns^	16	−1.983^*^	−5.302^**^
	Kerguelen Islands	34	8.5 ± 2.8	0.74 ± 0.12	0.76 ± 0.11	0.031^*^	16	−1.168^ns^	−3.147^*^
Antarctic prion	South Georgia	35	8.5 ± 2.6	0.69 ± 0.12	0.75 ± 0.13	0.075^**^	17	−1.773^ns^	−4.201^*^
	Kerguelen Islands	38	9.4 ± 3.5	0.71 ± 0.16	0.74 ± 0.16	0.047^ns^	15	−1.814^*^	−4.997^**^

*N*: number of individuals with reliable amplification. *A*: number of alleles (mean ± s.d.). *H*_*o*_: observed heterozygosity (mean ± s.d.). *H*_*e*_: expected heterozygosity (mean ± s.d.). *F*_*IS*_: inbreeding coefficient. *D*: Tajima’s statistic. *F*_*S*_: Fu’s statistic (* *P* < 0.05, ** *P* < 0.01, *** *P* < 0.001, ns: not significant)

Genetic structure was analysed using population-model based, and non-model based approaches. We used ARLEQUIN 3.5 [34] to perform model-free analyses of molecular variance (AMOVAs) and tested for significance using 1000 permutations. As a model based analysis, we used the Bayesian clustering algorithm implemented in STRUCTURE 2.3.4 [36]. We ran STRUCTURE under the admixture ancestry model and correlated allele frequencies without locality as priors. We explored values of K = 1–5 (10 iterations per K value), each consisting of 400,000 MCMC repetitions after a burn-in of 100,000. The most likely K value was determined using STRUCTURE HARVESTER [37] in accordance with the methods described in Evanno et al. [38]. Different iterations of the optimal K value were combined in CLUMPP 1.1.2 [39] and displayed using DISTRUCT 1.1 [40]. Genetic structure was further analysed using factorial correspondence analysis (FCA) of population multilocus scores carried out in GENETIX 4.05 [41]. Using the allele frequencies for all loci, FCA allows the visualisation in space of the genetic differences between individuals. We inferred gene flow in BAYESASS 3.0 using bi-directional migration rates (m) [42]. This method was successful in the study of immigration and emigration in populations that are not in equilibrium e.g. [4, 43, 44]. BAYESASS estimates the posterior probability of an individual’s history and allows an estimation of the rate and direction of recent dispersal [43]. The acceptance rates for the main parameters (i.e. ‘migration’ rate, inbreeding coefficient and allele frequencies) were adjusted during several preliminary runs. Convergence was assessed by checking the trace files in TRACER 1.6 [45]. Final parameter estimates were obtained after performing three independent runs using different starting random seed numbers. The MCMC was run for 50,000,000 iterations with a burn-in period of 10,000,000 and a sampling frequency of 5000 iterations.

### Genetic analyses using cytochrome b sequences

An 889-bp fragment of the cytochrome *b* (cyt *b*) gene was amplified using specific primers (CytB_Pri_F: 5′ -CTAGCTATACACTACACCGC-3′ and CytB_Pri_R: 5′ -CTAGTTGGCCGATGATGATG-3^′^) [15]. PCRs were conducted in 20 μl reaction volumes containing 100 ng DNA template, 10 mM of each primer, 10 mM dNTPs (Roth, Karlsruhe), 2 mM MgCl and 0.5 U Taq DNA polymerase (BioLabs Taq DNA polymerase) in a 1× PCR reaction buffer. Thermocycling included initial denaturation at 94 °C for 2 min, 30 cycles of denaturation at 94 °C for 30 s, annealing at 60 °C for 45 s and extension at 72 °C for 1 min, followed by a final extension step of 5 min at 72 °C. PCR products were purified by digestion with exonuclease-shrimp alkaline phosphatase (from Fermentas Life Sciences), following the manufacturer’s specifications. PCR products were then sequenced in both directions using Big Dye chemistry (Applied Biosystems) and run on an AB 3130xl genetic analyser (for cyt *b*). Sequences were assembled and aligned in CLC Main Workbench 6.9.2 (CLC bio, Aarhus, Denmark).

A Fairy prion *Pachyptila turtur* sample was included as an outgroup. The best-fit model of nucleotide substitution for each of our sequence alignment was determined using Bayesian Information Criterion scores and corrected Akaike Information Criterion values in MEGA 6.06 [46]. The cyt *b* trees were inferred by using the Maximum Likelihood method based on the Hasegawa-Kishino-Yano model [47] in MEGA, applying the Neighbor-Joining method for the heuristic search. Branch support was assessed with 10,000 pseudoreplicates (bootstrapping). Median-joining haplotype networks were estimated using NETWORK 5 [48]. Genetic structure was also investigated by AMOVA, calculating the variance component distributed between populations (*F*_ST_) following Weir & Cockerham [49] in ARLEQUIN 3.5 [34]. The significance of *F*_*ST*_ values were tested with 1000 permutations.

## Results

### Wintering range overlap

**Fig. 1:**
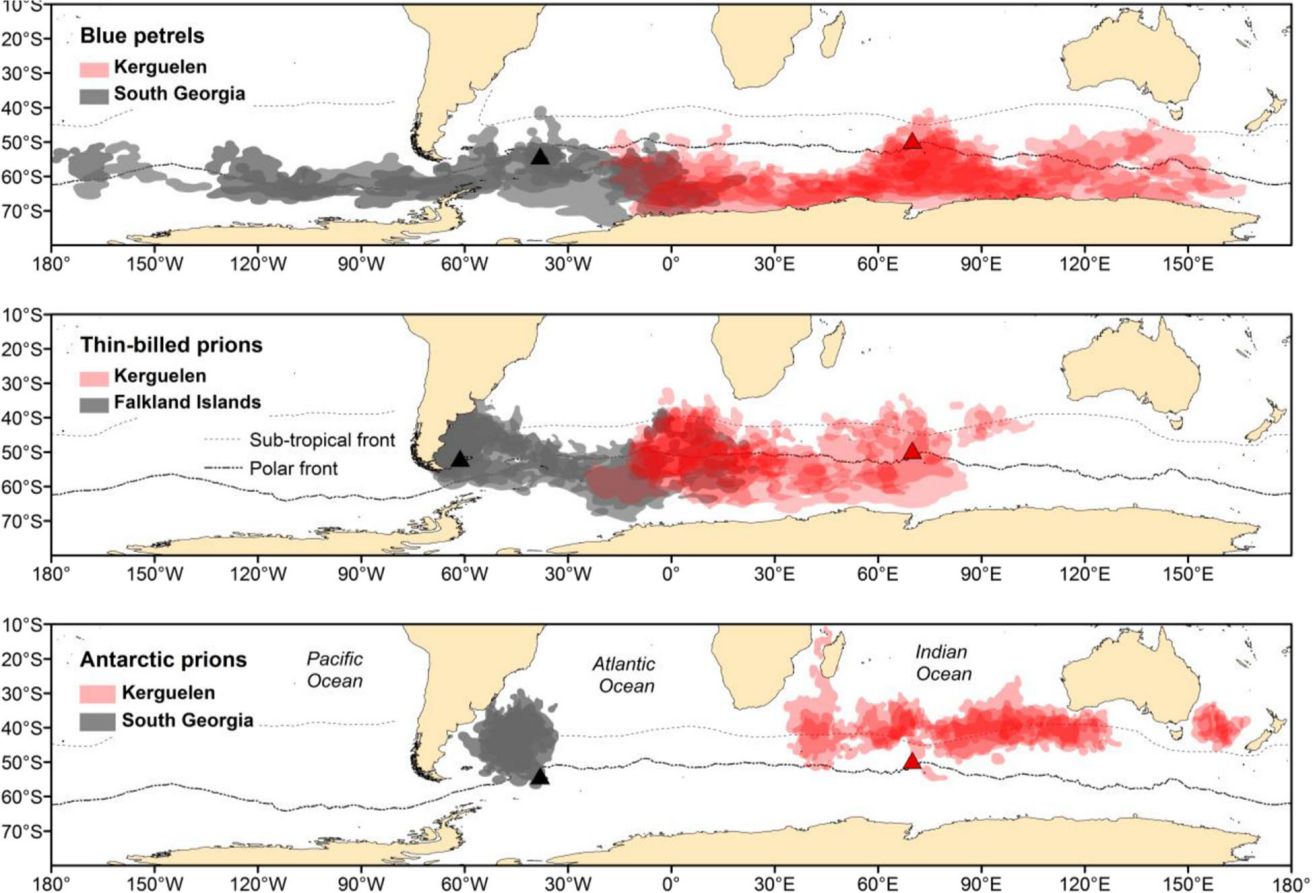
Winter (non-breeding) distributions of Atlantic and Indian Ocean populations of blue petrels *Halobaena caerulea*, thin-billed prions *Pachyptila belcheri* and Antarctic prions *P. desolata*, based on monthly 95% kernel distributions obtained using geolocators in austral winters 2010–2013 (for details, see [2, 26, 27]). *Black* and *red triangles* mark the Atlantic Ocean (Falklands/Malvinas, South Georgia) and Indian Ocean (Kerguelen) colonies, respectively

**Fig. 2:**
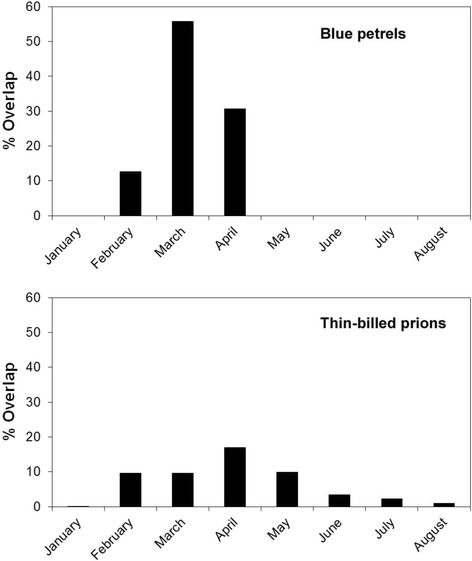
Overlap in winter (non-breeding distributions) of Atlantic and Indian Ocean populations, based on monthly 95% kernels, obtained using geolocators in austral winters 2010–2013. *Upper panel*: blue petrels *Halobaena caerulea*, *lower panel*: thin-billed prions *Pachyptila belcheri*

The non-breeding ranges of the tracked adult blue petrels from Kerguelen and South Georgia overlapped from February to April (Figs. 1 and 2; see Additional file 1: Figure S1), with the highest overlap, in March-April, of 56% (748,170 km^2^) of the overall distribution (1,343,647 km^2^) used by individuals of both populations. The ranges of the tracked adult thin-billed prions from Kerguelen and Falkland/Malvinas overlapped from February to August (Fig. 1), with the highest overlap, also in April, of 17% (1,448,000 km^2^) of the overall distribution (8,551,000 km^2^) used by individuals of both populations (see Additional file 1: Figure S1). The ranges of the tracked adult Antarctic prions from Kerguelen and South Georgia were >5000 km apart throughout the non-breeding season (see Fig. 1).

### Population genetic structure – microsatellite data

The inbreeding coefficient (*F*_*IS*_) based on 25 polymorphic microsatellites suggested a deviation from Hardy-Weinberg equilibrium due to an excess of homozygotes in all six populations. To test if this was due to non-random mating (either inbreeding or the existence of population genetic structure, i.e. the Wahlund effect [50]), or the existence of null alleles, we examined whether the effect was locus-specific. We found a significant deviation in seven of the 25 loci. Independently of the population analysed, always the same seven loci showed Hardy-Weinberg disequilibrium, which suggest the existence of null alleles. We therefore removed these seven loci from further analyses.

The genetic diversity found at the two major colonies of blue petrels, thin-billed prions, and Antarctic prions is summarised in Table 1. For most parameters considered, no differences were observed between populations from the Atlantic or Indian Oceans (Table 1). However, the inbreeding coefficient (*F*_*IS*_ *)* was higher in the Atlantic Ocean populations for all three species (Table 1).

**Fig. 3:**
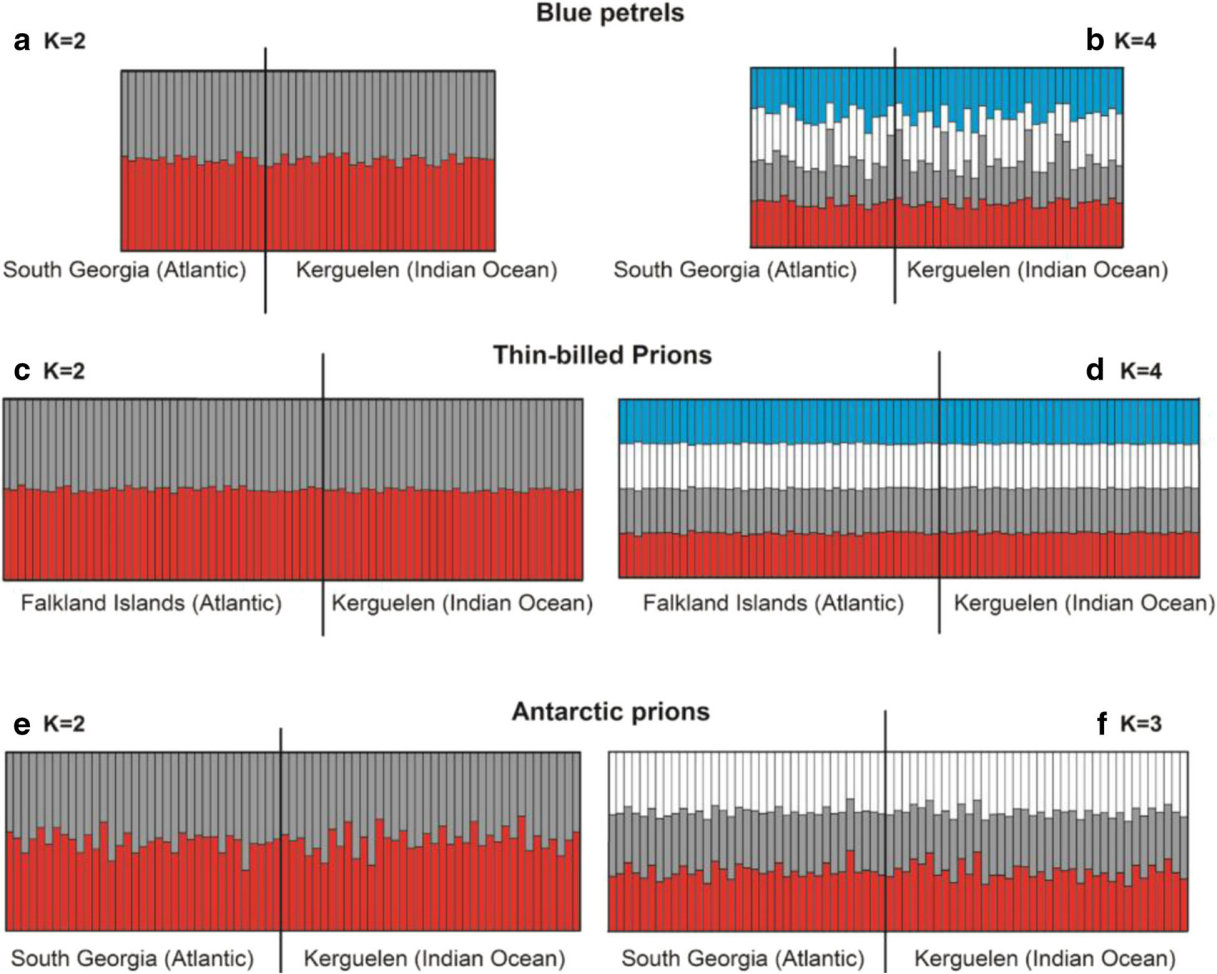
Results from cluster analysis of microsatellite data. The analyses were carried out on individuals from Atlantic and Indian Ocean populations of *blue* petrels *Halobaena caerulea*, thin-billed prions *Pachyptila belcheri* and Antarctic prions *P. desolata*, and are based on 18 microsatellite markers. The plots show the proportional membership of each individual in the genetic clusters (shown in different colours) calculated in STRUCTURE under K = 2 and the next most likely K (3 or 4), without priors

**Fig. 4:**
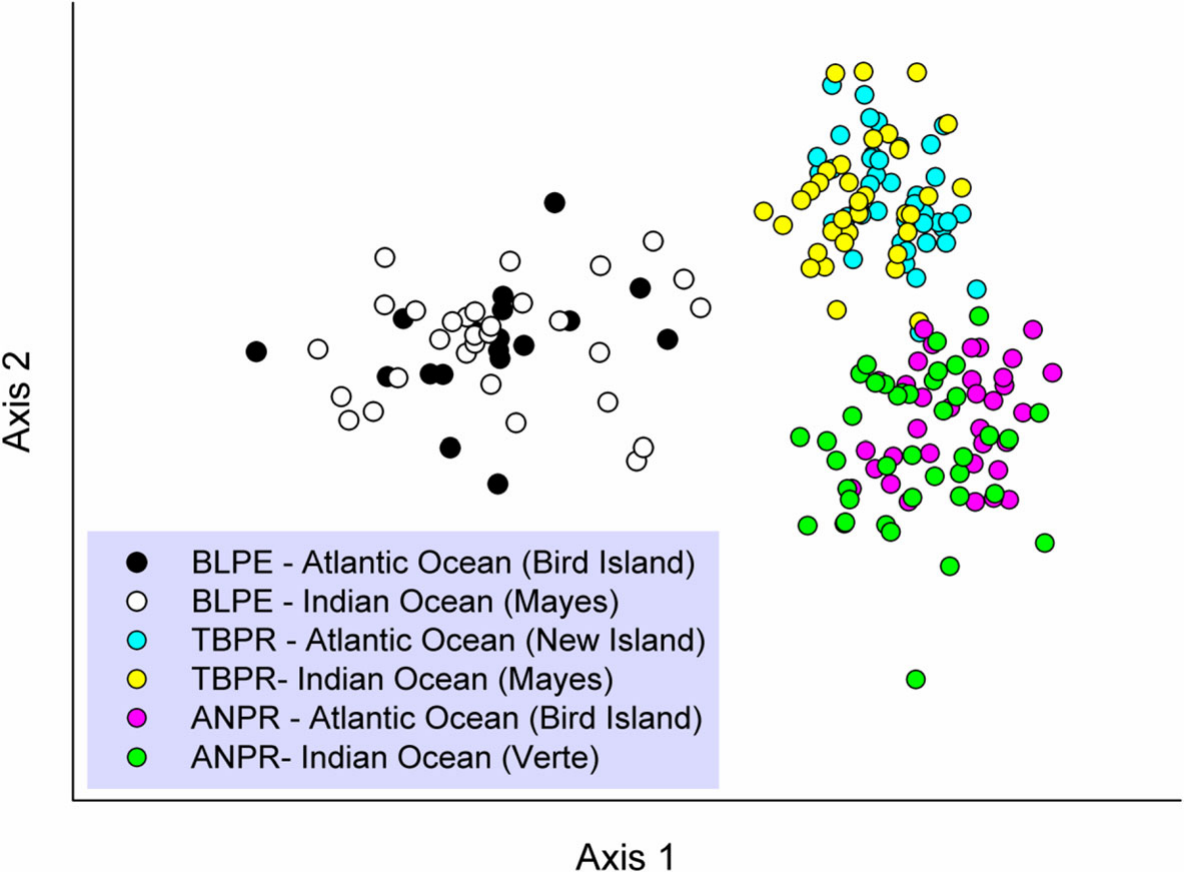
Genetic differentiation. The differentiation between Atlantic and Indian Ocean populations of *blue* petrels *Halobaena caerulea* (BLPE), thin-billed prions *Pachyptila belcheri* (TBPR), and Antarctic prions *P. desolata* (ANPR), is based on Factorial Correspondence Analysis (FCA) on 18 microsatellite markers

Bayesian clustering methods did not identify population genetic structure for any of the tested species. For all three species, the Evanno method estimated the highest likelihood LnPr(X|K) for K = 2. STRUCTURE also revealed homogenous distributions of individual genotypes, where all individuals were assigned to approximately equal proportions of the inferred clusters (Fig. 3). The graphical representation of genetic differentiation by FCA analyses similarly demonstrates the lack of clear population genetic structure within each species (Fig. 4; eigenvalue, *λ*, 1 and 2, and cumulative % of variability explained: blue petrels, *λ*_*1*_ = 0.183, *λ*_*2*_ = 0.159, 12.6%; Antarctic prions, *λ*_*1*_ = 0.134, *λ*_*2*_ = 0.123, 9.8%; thin-billed prions, *λ*_*1*_ = 0.113, *λ*_*2*_ = 0.109, 9.9%).

In addition, classical estimators of population differentiation were very low between Atlantic and Indian Ocean populations for all species. AMOVA results indicated a higher population differentiation between the populations of Antarctic prions (*F*_*ST*_ = 0.008, *P* < 0.001), than those of blue petrels (*F*_*ST*_ = 0.001, *P* < 0.001), and thin-billed prions (*F*_*ST*_ = −0.0002, *P* = 0.002).

Bi-directional migration rates showed differences in the net direction of gene flow between Indian and Atlantic Ocean populations. The blue petrels and Antarctic prions tended to migrate significantly more from the Indian to Atlantic Oceans, whereas thin-billed prions migrated mainly in the opposite direction (Table 2).

**Table 2:** Posterior mean migration rates and standard deviation of the marginal posterior distribution for each estimate. Mean migration rates (m) as a proportion from 0 to 1 and standard deviation (s.d.) were calculated between Atlantic and Indian Ocean populations of the blue petrel *Halobaena caerulea*, thin-billed prion *Pachyptila belcheri*, and Antarctic prion *P. desolata*

Species		m[Atlantic] ← [Indian]	m[Indian] ← [Atlantic]
Blue petrel	m	0.317	0.011
	s.d.	0.015	0.011
Thin-billed prion	m	0.008	0.324
	s.d.	0.009	0.009
Antarctic prion	m	0.324	0.009
	s.d.	0.009	0.009

Notes: m[Atlantic] ← [Indian]: is the fraction of individuals in the Atlantic Ocean population that are migrants derived from the Indian Ocean population (per generation); m[Indian] ← [Atlantic]: is the fraction of individuals in the Indian Ocean population that are migrants derived from the Atlantic Ocean population (per generation)

### Population genetic structure – cytochrome b sequences

**Fig. 5:**
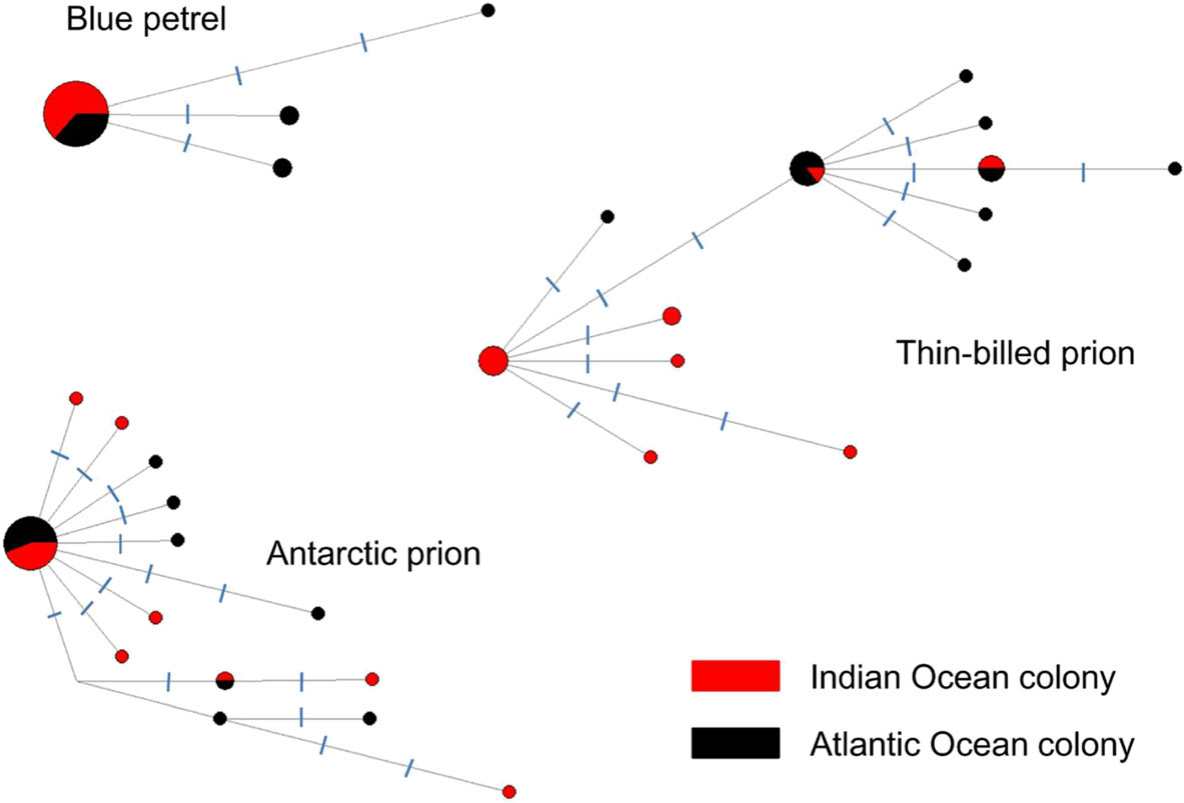
Median-joining networks. The networks are based on cytochrome *b* sequences from individuals belonging to Atlantic and Indian Ocean populations of blue petrels *Halobaena caerulea*, thin-billed prions *Pachyptila belcheri* and Antarctic prions *P. desolata*. The size of the *circles* is proportional to haplotype frequency. Hash-marks crossing line connections represent mutational steps

The median-joining haplotype networks were star-shaped, in that several less-frequent haplotypes were closely related to a single common haplotype (Fig. 5). The most common haplotype in blue petrels (isolated in 22 individuals) was present in both the Atlantic and Indian Ocean colonies (Fig. 5). Interestingly, this was the only haplotype present in the Indian Ocean colony, and three others were present only in the Atlantic (Fig. 5). Similarly, in the Antarctic prion, the most common of the 14 haplotypes was shared by both populations (Fig. 5). In contrast, the 13 haplotypes in the thin-billed prion were reasonably well structured into Atlantic and Indian Ocean haplotype groups, but also with a degree of sharing between populations.

High between-population haplotype sharing was also inferred from maximum likelihood reconstructions of species-level phylogenies in blue petrels and Antarctic prions, where all splits included individuals from both colonies and with the highest bootstrap support being 69% (see Additional file 1: Figures S2 to S4). Again the cyt *b* phylogeny for thin billed prions appeared more structured, with a lower degree of haplotype sharing between populations.

High haplotype sharing between Atlantic and Indian Ocean populations resulted in small non-significant between-population (*F*_ST_) values in blue petrels, and small but significant between-population values in Antarctic prions. However, the more structured cyt *b* sequences between thin-billed prion populations were manifested as larger and significant *F*_ST_ values using AMOVA (Table 3).

**Table 3:** Results of the AMOVA testing for genetic structure in the cytochrome *b* sequences. Test were carried out between Atlantic and Indian Ocean populations of the blue petrel *Halobaena caerulea*, thin-billed prion *Pachyptila belcheri*, and Antarctic prion *P. desolata*

Species	Source of variation	*d.f.*	Sum of squares	Percentage of variation	*F*_*ST*_
Blue petrel	Between populations	1	0.280	2.3	0.022^ns^
	Within populations	25	5.350	97.7	
Thin-billed prion	Between populations	1	7.148	35.1	0.351^***^
	Within populations	25	21.543	64.9	
Antarctic prion	Between populations	1	0.477	2.7	0.027^**^
	Within populations	29	23.265	97.3	

*P < 0.05, ** *P* < 0.01, *** *P* < 0.001, ns: not significant

## Discussion

In the present study, we compare the population genetic structure of three small pelagic petrels in relation to their distribution during the non-breeding season. The three petrels differed in the degree of spatio-temporal segregation of birds from large populations in the Atlantic and Indian Oceans, ranging from no overlap in Antarctic prions, limited spatial overlap for a prolonged period (6 months, max. 17%) in thin-billed prions, to more pronounced overlap but for a shorter time (3 months, max. 56%) in blue petrels. Based on this and on the hypothesis of Friesen et al. [4], we would expect the least gene flow in the Antarctic prions.

In contrast, high levels of gene flow in all three species meant that these differences in relative segregation of non-breeding ranges were barely detectable through standard Bayesian and multivariate analyses of population genetic structure. Only model-free analyses of hierarchical genetic structure through AMOVA indicated low, but significant levels of population differentiation in both nuclear and mitochondrial marker sets between Atlantic and Indian Ocean populations of Antarctic prions, in line with expectations. In the blue petrel, which showed the highest spatial overlap between populations in the non-breeding season, there was very little between-population differentiation at both molecular levels, which was also in line with our hypotheses.

The lack of population genetic structure of the species studied here is similar to results observed in other Southern Ocean seabirds that feed in pelagic waters e.g. [8, 9]. The Procellariiformes have adapted to pelagic foraging, and only come ashore to breed, usually on remote islands [51]. In the Southern Ocean, there are relatively few island groups suitable for breeding, and a pronounced genetic differentiation between populations might be expected based on the large distances and strong natal philopatry e.g. [52–54]. Alternatively, as these seabirds are highly mobile, they could maintain high levels of gene flow, as suggested by our results. The high migration rates also suggest that natal philopatry is not as strong as previously thought. In the breeding season, petrels are central-place foragers, constrained in their foraging distribution by the necessity to return to their nest site at regular intervals to incubate the egg or feed the chick. Even so, they can exploit vast ocean areas, foraging >1000 km from the nest [13, 28, 55]. In the non-breeding season, when not restricted by breeding duties, many seabird species, including the Procellariiformes, move over even larger distances, sometimes making circumpolar or trans-equatorial migrations [56–58]. The distribution of prions and blue petrels at sea has been the focus of recent stable isotope and tracking studies during the breeding and non-breeding season, which indicate considerable ecological segregation [2, 16, 17, 19, 59]. This segregation may have led to the speciation of prions and blue petrels via environmental specialisation (e.g. [60]) as reflected by genetic differences among species (Fig. 4). Other seabird species complexes breeding in different ecological (latitudinal) ranges in the Southern Ocean show a similar pattern (e.g. great albatrosses *Diomedea* spp. [61]; northern and southern rockhopper penguins *Eudyptes moseleyi* and *E. chrysocome* [62]).

Within our three study species, we found considerable admixture and evidence for high migration rates (Table 2). The lack of population structure even in Antarctic prions with non-overlapping wintering areas implies a higher gene flow than expected from the observed non-breeding distributions of adult birds. This may reflect the greater dispersal of young birds, which may spend several years roaming the oceans before settling to breed and may be much more wide-ranging even than adults during the non-breeding season [63]. Ecological studies on the role of spatial structure in determining the dynamics of populations suggest that migration rates below 0.10 (10% of the individuals between the populations each generation) are indicative of populations behaving independently [64]. In the three studied species, in addition to the high migration rates, BAYESASS analyses indicated a source-sink relationship between populations, with migration in one direction being much higher than in the opposite (Table 2). The reason for these asymmetries would require further detailed study; possible mechanisms include subtle differences in mating behaviour, low natal philopatry, or an active selection of the most productive colony by pre-breeders prior to recruitment. However, chick-provisioning rates observed for thin-billed prions do not support the latter hypothesis: although chicks on Kerguelen (Indian Ocean) are fed less frequently than those in the Falklands/Malvinas [65], the former received over 30% of immigrants per generation. To understand the reason for the observed asymmetries in the patterns of gene flow across ocean basins, a better understanding of the movements of juveniles and immatures prior to pair formation would be needed. However, prions only start breeding at 4–7 years old [66], and multi-year remote tracking is not yet feasible in these very small seabirds.

The mitochondrial data in the present study also allow an analysis of population demographic history. Negative Fu’s *F*_*S*_ for thin-billed and Antarctic prions, as well as Tajima’s *D* for thin-billed prions at the Falklands and Antarctic prions at Kerguelen (Table 1), and the star-shaped networks may be considered as signatures of recent population expansions (Fig. 5). Natural climate cycles, such as past glacial/interglacial phases, shape species distributions. Many Southern Ocean species have undergone range restrictions and expansions associated with glacial cycles [67]. The suitable glacial refugia for seabirds in the subantarctic during glacial maxima were restricted to a few lower-latitude islands (Gough Island, the Falkland Islands, or around New Zealand) [67, 68]. The patterns observed in the cytochrome *b* networks in the prions and blue petrels are compatible with a restriction to glacial refugia and subsequent population expansions when the species recolonized other subantarctic islands. A similar scenario has been postulated for other Southern Ocean seabirds, including king penguins *Aptenodytes patagonicus* [68], Emperor penguins *Aptenodytes forsteri* [69] and pygoscelid penguins [70].

## Conclusions

In summary, our study highlights the low genetic structure of three abundant pelagic seabirds which show varying degrees of spatio-temporal segregation at sea between different breeding populations. In line with several other Southern Ocean seabirds, geographic distance between breeding colonies in these petrels does not generate genetic differences, regardless of the degree of overlap in non-breeding distributions. Thus, our study did not support the hypothesis of Friesen et al. [4] who suggested that the extent of genetic structure in seabirds could be explained by the overlap of non-breeding areas. Contrary to other seabird species with two or more population-specific non-breeding areas, the three small petrels in our study did not show population genetic structure. Some of the species investigated in [4] differ from this study either in being resident at, or near, breeding colonies or having passed through population bottlenecks. The reasons for other species differences (e.g. differing degrees of philopatry) need further investigation.

## Additional file

Additional file 1: Figure S1. Distribution of Atlantic and Indian Ocean populations of blue petrels *Halobaena caerulea* and thin-billed prions *Pachyptila belcheri* during the period of highest overlap in the winter. **Figure S2.** Cytochrome *b* gene tree for the two major populations in the Atlantic and Indian Oceans of the blue petrel *Halobaena caerulea*. **Figure S3.** Cytochrome *b* gene tree for the two major populations in the Atlantic and Indian Oceans of the thin-billed prion *Pachyptila belcheri*. **Figure S4.** Cytochrome *b* gene tree for the two major populations in the Atlantic and Indian Oceans of the Antarctic prion *Pachyptila desolata*. (PDF 2600 kb)
