# Supplementary material for: Does genetic structure reflect differences in non-breeding movements? A case study in small, highly mobile seabirds
Source: BMC Evol Biol. 2017 Jul 5;17:160. doi: 10.1186/s12862-017-1008-x (PMC5499058; doi:10.1186/s12862-017-1008-x)

Additional file 1

Authors:

Petra Quillfeldt, Yoshan Moodley, Henri Weimerskirch, Yves Cherel, Karine Delord, Richard Phillips, Joan Navarro, Luciano Calderón, & Juan F. Masello

Title:

Does the genetic structure reflect differences in non-breeding movement? A case study in small, highly mobile seabirds

Figure S1. Distribution of Atlantic and Indian Ocean populations of blue petrels *Halobaena caerulea* and thin-billed prions *Pachyptila belcheri* during the period of highest overlap in the winter.

Figure S2. Cytochrome *b* gene tree for the two major populations in the Atlantic and Indian oceans Oceans of the blue petrel *Halobaena caerulea*.

Figure S3. Cytochrome *b* gene tree for the two major populations in the Atlantic and Indian oceans Oceans of the thin-billed prion *Pachyptila belcheri*.

Figure S4. Cytochrome *b* gene tree for the two major populations in the Atlantic and Indian oceans Oceans of the Antarctic prion *Pachyptila desolata*.

Figure S1. Distribution of Atlantic and Indian Ocean populations of Blue petrels *Halobaena caerulea* and Thin-billed prions *Pachyptila belcheri* during the highest overlap in winter distribution, based on monthly 95% kernel distributions, obtained by geolocator tracking in the austral winters 2010-2013 (for details, see [2,17,18,27]). Black triangles mark the breeding colonies of Atlantic Ocean populations, and red triangles mark the Kerguelen archipelago, the site of the breeding colonies in the Indian Ocean.

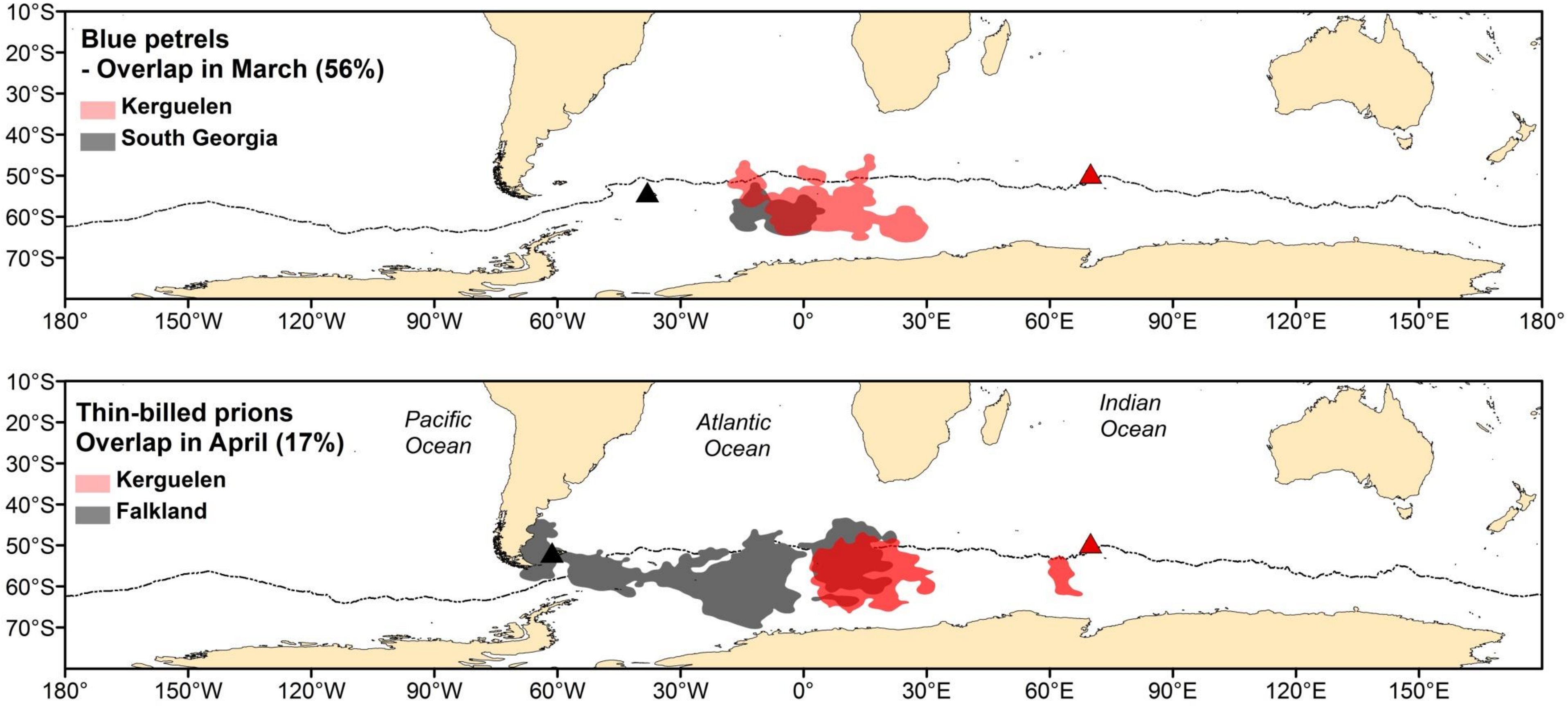

Figure S2 Cytochrome *b* gene tree for the two major populations in the Atlantic and Indian oceans of the Blue petrel *Halobaena caerulea*, inferred by using the maximum likelihood method based on the Hasegawa-Kishino-Yano model and applying the Neighbor-Joining method for the heuristic search. The name used for the individual samples codes the individual name (two or three digit number), the species (here “c” for *caerulea*) and the colony of origin (MY: Île Mayès, Kerguelen, Indian Ocean, in red in the bar to the right; BI: Bird I., South Georgia; Atlantic Ocean, in black in the bar). A Fairy prion *Pachyptila turtur* sample from Beauchêne I., Falkland / Malvinas Is. (289tBE; 52°55′S, 59°11′W) was used as out-group.

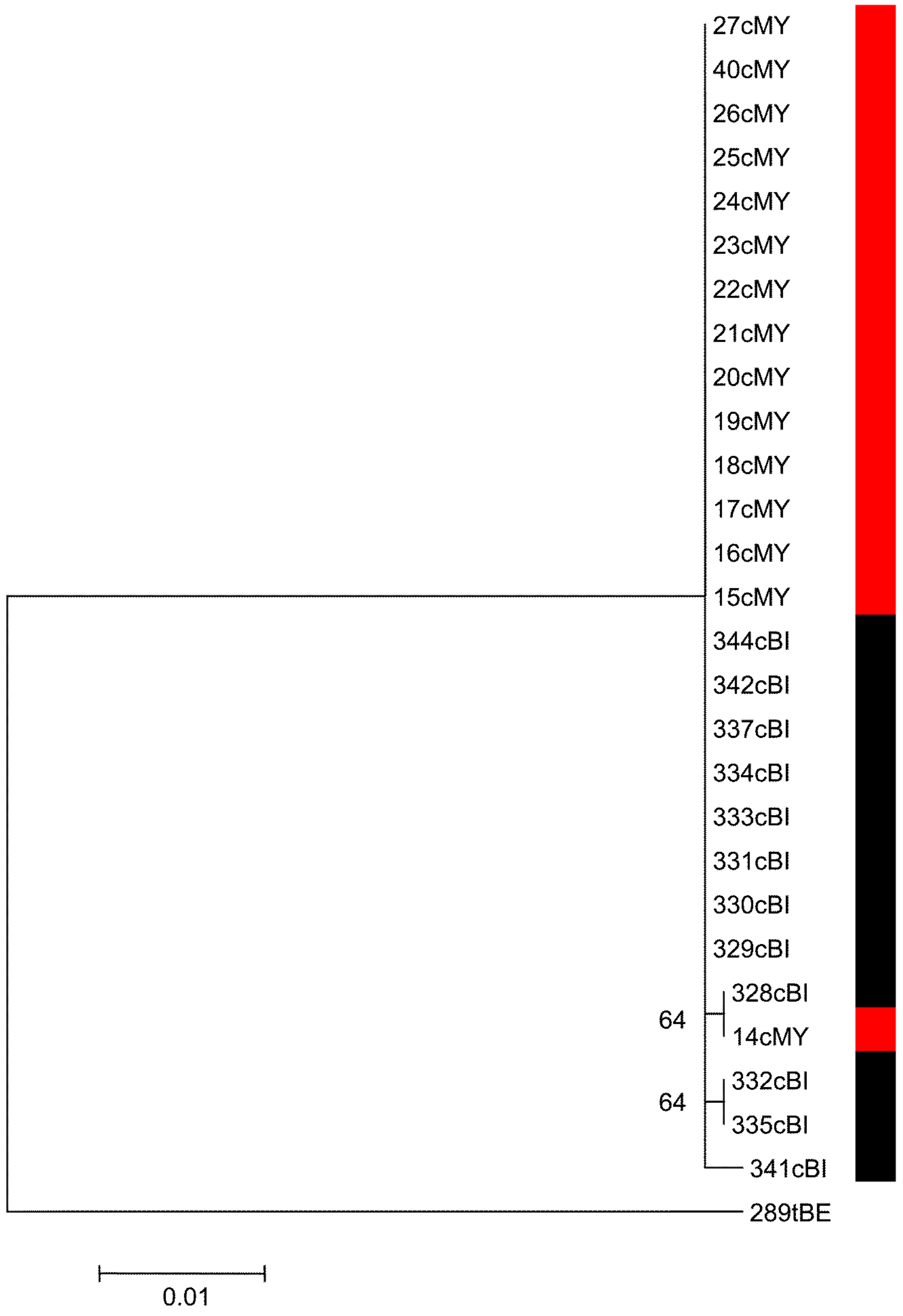

Figure S3. Cytochrome *b* gene tree for the two major populations in the Atlantic and Indian oceans of the Thin-billed prion *Pachyptila belcheri*, inferred by using the maximum likelihood method based on the Hasegawa-Kishino-Yano model and applying the Neighbor-Joining method for the heuristic search. The name used for the individual samples codes the individual name (two or three digit number), the species (here “b” for *belcheri*) and the colony of origin (MY: Île Mayès, Kerguelen, Indian Ocean, in red in the bar to the right; NI: New Island, Falkland/Malvinas Islands, Atlantic Ocean, in black in the bar). A Fairy prion *Pachyptila turtur* sample from Beauchêne I., Falkland / Malvinas Is. (289tBE; 52°55′S, 59°11′W) was used as out-group.

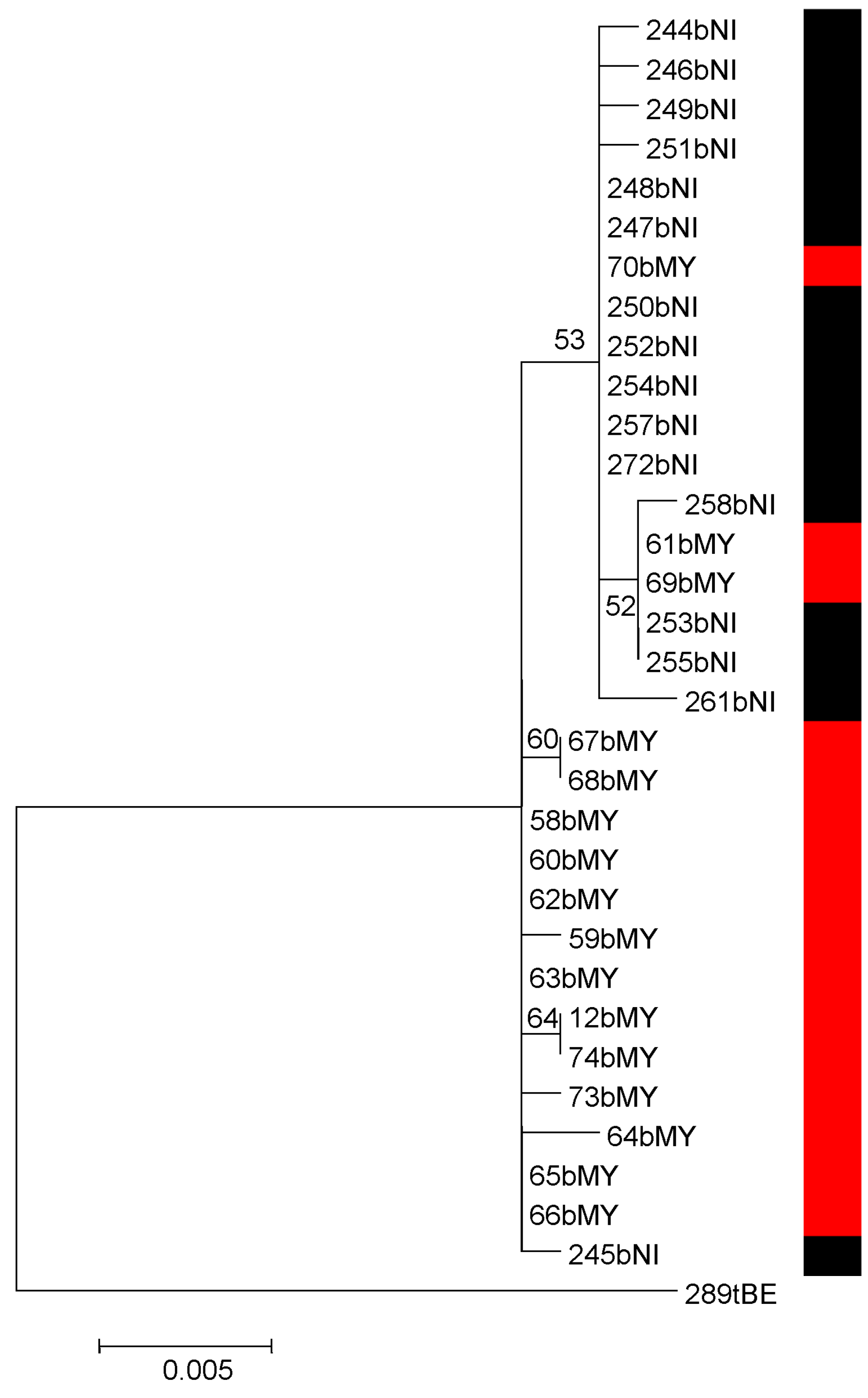

Figure S4. Cytochrome *b* gene tree for the two major populations in the Atlantic and Indian oceans of the Antarctic prion *Pachyptila desolata*, inferred by using the maximum likelihood method based on the Hasegawa-Kishino-Yano model and applying the Neighbor-Joining method for the heuristic search. The name used for the individual samples codes the individual name (two or three digit number), the species (here “d” for *desolata*) and the colony of origin (VE: Île Verte, Kerguelen, Indian Ocean, in red in the bar to the right; BI: Bird I., South Georgia, Atlantic Ocean, in black in the bar). A Fairy prion *Pachyptila turtur* sample from Beauchêne I., Falkland / Malvinas Is. (289tBE; 52°55′S, 59°11′W) was used as out-group.

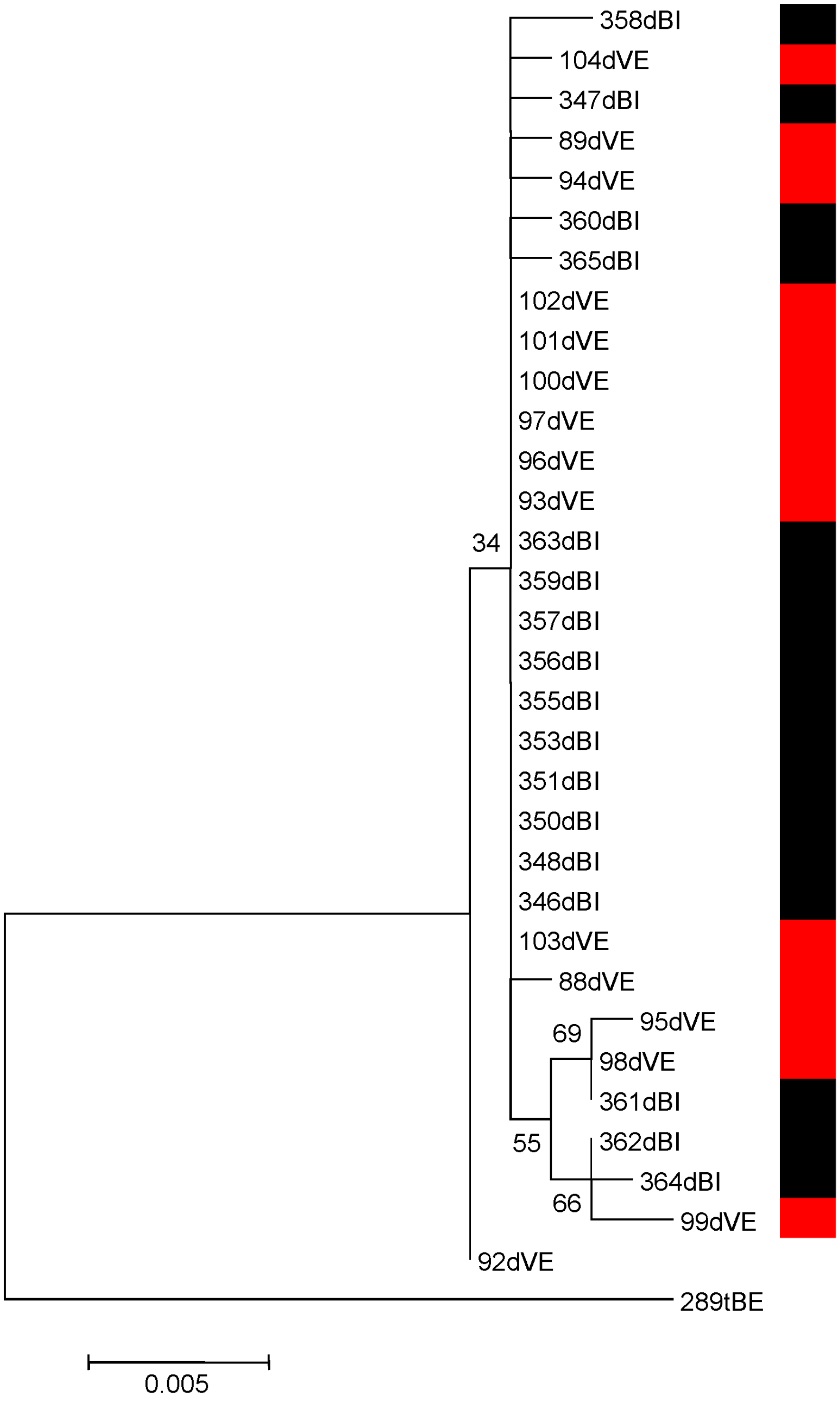

Supplement: Additional file 1: Figure S1. — Distribution of Atlantic and Indian Ocean populations of blue petrels Halobaena caerulea and thin-billed prions Pachyptila belcheri during the period of highest overlap in the winter. Figure S2. Cytochrome b gene tree for the two major populations in the Atlantic and Indian Oceans of the blue petrel Halobaena caerulea. Figure S3. Cytochrome b gene tree for the two major populations in the Atlantic and Indian Oceans of the thin-billed prion Pachyptila belcheri. Figure S4. Cytochrome b gene tree for the two major populations in the Atlantic and Indian Oceans of the Antarctic prion Pachyptila desolata. (PDF 2600 kb) [file 12862_2017_1008_MOESM1_ESM.pdf]
